# Self-Medication during and after Cancer: A French Nation-Wide Cross-Sectional Study

**DOI:** 10.3390/cancers15123190

**Published:** 2023-06-15

**Authors:** Julie Maraud, Sabrina Bedhomme, Bruno Pereira, Sophie Trévis, Marine Jary, David Balayssac

**Affiliations:** 1 UFR de Pharmacie, Université Clermont Auvergne, F-63000 Clermont-Ferrand, France; julie.maraud@etu.uca.fr; 2 UR ACCePPT, Université Clermont Auvergne, F-63000 Clermont-Ferrand, France; sabrina.bedhomme@uca.fr; 3 Direction de la Recherche Clinique et de l’Innovation, CHU Clermont-Ferrand, F-63000 Clermont-Ferrand, France; bpereira@chu-clermontferrand.fr; 4 Pharmacie, CHU Clermont-Ferrand, F-63000 Clermont-Ferrand, France; strevis@chu-clermontferrand.fr; 5 Chirurgie et Oncologie Digestive, CHU Clermont-Ferrand, F-63000 Clermont-Ferrand, France; mjary@chu-clermontferrand.fr; 6 UMR1107, NEURO-DOL, INSERM, Direction de la Recherche Clinique et de l’Innovation, Université Clermont Auvergne, CHU Clermont-Ferrand, F-63000 Clermont-Ferrand, France

**Keywords:** cancer, self-medication, health-related quality of life, symptoms

## Abstract

**Simple Summary:**

Self-medication by patients is an underassessed topic in the field of cancer. This French observational study aimed to assessed self-medication practices, perceived risks, and the relation with symptoms and quality of life in cancer patients and survivors. Half of the patients declared practicing self-medication. Dietary supplements and pain medications were the main products used for self-medication. Self-medication was practiced in order to manage the adverse effects of anticancer therapies by two-thirds of patients, and by half of them to improve the efficacy of anticancer therapies. Most patients were very confident with the safety of self-medication. Self-medication practices were associated with altered social functioning, pain, insomnia, and financial difficulties. Finally, in these cancer patients and survivors, self-medication practices could evidence the undermanagement of cancer and treatment-related adverse effects.

**Abstract:**

(1) Background: Little data are available in Western countries regarding self-medication practices in the context of cancer. The aim of this study was to describe the prevalence of self-medication practices during (cancer patients) and after cancer (cancer survivors). (2) Methods: This multicenter, cross-sectional, and online study was designed to assess self-medication prevalence. Other objectives were explored, notably the medication types, the perceived risks, and the relation with symptoms and quality of life. (3) Results: Among the 518 patients analyzed, 56.4% declared they practiced self-medication. Dietary supplements and pain medications were used by more than half of the patients. Self-medication was practiced in order to manage the adverse effects of anticancer therapies (63.8%), for which pain was the leading indication (39%), and to improve the efficacy of anticancer therapies (43.8%, cancer patients). Patients believed that self-medication could not lead to drug interactions with anticancer therapies (84.9%, cancer patients), or to adverse effects (84.6%, cancer patients and survivors). Self-medication practices were associated with altered social functioning, pain, insomnia, and financial difficulties. (4) Conclusions: Self-medication was performed by more than half of the responders (ongoing or past cancer) and could be a marker of the undermanagement of cancer and treatment-related adverse effects.

## 1. Introduction

The number of new cases of cancer worldwide in 2020 (excluding non-melanoma skin cancer) was estimated at about 18,094,716 [1], and 382,000 in France in 2018, [2]. Breast, lung, colorectal, and prostate cancers are by far the most frequent cancers, both worldwide and in France [1,2]. Although there are strong disparities between the incidence and type of cancers worldwide, cancer is one of the main issues of public health [3].

According to the World Health Organization (WHO), “self-medication involves the use of medicinal products by the consumer to treat self-recognized disorders or symptoms, or the intermittent or continued use of a medication prescribed by a physician for chronic or recurring diseases or symptoms” [4]. In France, self-medication is defined very similarly, i.e., “self-medication is the use of a drug by a person on their own initiative, for the treatment of simple and already known symptoms or mild illnesses” [5]. However, there is not a consensus on the definition of self-medication. As proposed by Baracaldo-Santamaría et al., self-medication should be regarded “as a process in which OTC and prescription medications, herbal products, home-made remedies, nutritional supplements and vitamins are purchased, selected, used, and consumed; it is a behavior in which the individual aims to treat self-recognized disorders or symptoms 48 or tries to alleviate third parties’ ailments” [6].

Self-medication is increasingly popular worldwide, with an estimated prevalence of 67% (95% confidence interval—95 CI: 62; 73), and with a strong heterogeneity worldwide [7]. In older European adults, prevalence of self-medication ranges from 13.3% in Spain [8] up to 57% in Serbia [9]. In the case of Spain, a statistically significant increase in the prevalence of self-medication has been observed, from 7.79% in 2009 to 13.3% in 2014 [8].

As described by the WHO, self-medication may provide several benefits at the individual, social, and economic levels (e.g., efficacy; quick and available medicines; active role and self-reliance of patients; educational opportunities; convenience; saving medical resources from being wasted on minor conditions; lowering the costs of community-funded health care; reducing absenteeism from work due to minor symptoms, etc.). However, self-medication can be associated with several potential risks for the individual consumer (e.g., incorrect self-diagnosis; incorrect choice of therapy; failure to recognize special pharmacological risks; rare but severe adverse effects; failure to recognize or self-diagnose contraindications, interactions, warnings and precautions; failure to report current self-medication to the prescribing physician (risk of double medication or harmful interaction); failure to recognize or report adverse drug reactions; incorrect route or manner of administration; inadequate or excessive dosage, etc.) [4]. Self-medication practices can lead to safety issues, such as hospital admissions for adverse drug reactions due to inappropriate self-medication or misuse by patients (11.6%) [10].

Because cancer and cancer management lead to several symptoms and iatrogenic effects, cancer is a specific condition that can promote self-medication practices. Regarding the use of complementary and alternative medicine (CAM), which includes not only active product-based strategies but also mind–body interventions, energy therapies, and manipulative and body-based therapies [11], as many as 87% of individuals with cancer, out of 17,639 patients in the USA, reported at least one form of CAM therapy following their diagnosis [11]. A population-based study showed that cancer survivors were more likely to use CAM therapies than individuals without cancer, with 65% reporting CAM use in their lifetime and 43% having used these therapies in the past year, compared with 52% and 37%, respectively, among non-cancer individuals (*p* < 0.001) [12]. Reasons for using CAM by cancer patients have been revealed, such as increasing the capacity to fight the cancer, improving their physical and emotional well-being [13], while for cancer survivors these reasons included wellness, immune function, pain-related symptoms, improving medical treatment, costs of medical treatment, or based on recommendations by providers and close neighbors [12]. However, here again, self-medication practices by cancer patients can lead to serious adverse events (e.g., opium poisoning to treat radiodermatitis [14], oleander poisoning to prevent/treat a possible malignant thyroid disease [15], or hydrazine sulfate poisoning to treat a squamous-cell carcinoma of the maxillary sinus [16]). However, few data on self-medication in the context of cancer are available in Western countries, regarding the practices, the products used, the perceived risks, and the symptoms.

The aim of this study was to describe the prevalence of self-medication practices during and after cancer. Secondary objectives included the perceived risk of the self-medication practices, and the relation between self-medication and patient health-related quality of life (HRQoL), and symptoms.

## 2. Materials and Methods

### 2.1. Study Design

This multicenter, observational, and cross-sectional study was designed to assess the practices of self-medication of patients during and after cancer. The primary objective was the assessment of self-medication prevalence during (cancer patients) cancer and after the end of treatment (cancer survivors). The secondary objectives were the description of the products used (types of products, indications declared by the patients, place of purchase, council on self-medication, and disclosure to health professionals of self-medication practice), the self-medication-related risks perceived by the patients, and the relation between the self-medication and patients’ characteristics, cancer type, symptoms, and HRQoL. Patients were assessed once, and no longitudinal assessment was performed.

The study was designed according to the STROBE (Strengthening the Reporting of Observational Studies in Epidemiology) guidelines [17]. The study protocol was registered on the ClinicalTrials.gov website NCT05156931. The study was anonymous and approved by a local ethics committee (Comité de protection des personnes Ile de France II, No. 2021-A00228-41, 1 December 2021). Each participant’s informed consent was obtained together with their answers to the survey.

### 2.2. Setting

This study was conducted in France with the help of 11 French associations of cancer patients (details of associations: Supplementary Materials File S1) and 27 Facebook pages (details of Facebook pages: Supplementary File S1). The estimated number of patients that were members of these patients’ associations and Facebook pages was about 7549 individuals (representing a panel of patients). The inclusion of patients and data collection were carried out from 14 December 2021 until 26 April 2022.

### 2.3. Participants

In this panel of patients, inclusion criteria were patients aged ≥18 years, male or female, declaring that they were being treated or had been treated for a cancer. Exclusion criteria were patients not speaking French, or living outside France, or persons supporting cancer patients. Eligible patients were contacted by email via French associations of cancer patients.

### 2.4. Variables

The primary endpoint was the self-medication practice (yes/no) “Do you currently practice self-medication and/or do you use products to improve your health?” The definition of self-medication was mentioned in the questionnaire, and described as follows according to the French National Health Insurance: “self-medication is the use of a drug by a person on their own initiative, for the treatment of simple symptoms and already known, or benign diseases” [5]. As defined below, for self-medication, we have mentioned all therapeutic strategies that can be used on the patient’s own decision, including OTC medications, herbal medicines, aromatherapy medicines (essential oils), homeopathy medicines, and dietary supplements.

The secondary endpoints were:

- The modalities: over-the-counter (OTC) pain medication, OTC digestive tract medication, OTC anxiety and sleep disorder medications, OTC mouth and throat medications, OTC venous disorder medications, herbal medicines, aromatherapy medicines, homeopathy medicines, and dietary supplements;
- The indication for the self-medication declared by the patients (management of adverse effects (yes/no; if yes for anxiety/stress, depression, hair/nail disorders, hot flush, constipation, diarrhea, nausea/vomiting, stomach aches, pain, fatigue, mouth disorders, skin disorders, vaginal disorders, sleep disorders, breath disorders, sexual disorders, or others) and/or improvement of anticancer therapies (yes/no));
- The place of purchase (pharmacy, mall, internet, or other);
- The council received for self-medication (oncologist, general practitioner, pharmacist, friend, family, internet, press, or other);
- The disclosure to health professionals involved in cancer management of self-medication practice (oncologist, general practitioner, pharmacist, other, or none);
- The perceived risks related to the self-medication (drug interactions and adverse effects; yes/no);
- The symptoms and HRQoL were assessed with the QLQ-C30 questionnaire [18]. The scoring of the QLQ-C30 questionnaire was carried out according to EORTC recommendations (https://qol.eortc.org/questionnaire/eortc-qlq-c30 (accessed on 6 September 2021)). The QLQ-C30 was divided into 3 subscales with a global health status (0 worst to 100 best), the functional scales (0 worst to 100 best for physical, role, emotional, cognitive, and social functioning), and the symptom scales (0 least to 100 worst for fatigue, nausea and vomiting, pain, dyspnea, insomnia, appetite loss, constipation, diarrhea and financial difficulties);
- The oncological characteristics of patients (ongoing or past cancer and localization, ongoing or past treatments);
- The socio-demographic characteristics of patients (sex, age, weight, height, French department of residence, monthly income, and education).

### 2.5. Data Sources/Measurement

All the data were obtained from the completed online questionnaires. All the data were recorded and managed using REDCap^TM^ electronic data capture tools hosted at the University Hospital of Clermont-Ferrand [19].

### 2.6. Study Size

Based on the literature, it was expected that the proportion of self-medication practice would be about 25–30% in this population of cancer patients and survivors [13,20]. A sample size of 800 patients was determined to ensure that the 95 CI of self-medication prevalence had an accuracy of 3–4%.

### 2.7. Statistical Methods

Continuous data were expressed as mean and standard deviation or by median and interquartile range. The assumption of normality was studied using the Shapiro–Wilk test. The continuous data were compared between independent groups (such as variable no self-medication vs. self-medication for all patients, and variable for cancer survivors and for cancer patients) using Student *t*-test or the Mann–Whitney test when the assumptions of *t*-test were not met. The assumption of homoscedasticity was studied using the Bartlett test. When appropriate (omnibus *p*-value for ANOVA or Kruskal–Wallis less than 0.05), post hoc tests for multiple comparisons were performed to take into account type I error correction: Tukey–Kramer test after ANOVA or Dunn’s test post-Kruskal–Wallis. The comparisons between groups concerning categorical data (such as education level and type of cancer) were performed using Chi-squared or Fisher’s exact tests, followed when appropriate by Marascuilo’s procedure. To determine factors associated with self-medication practices, multivariable analyses were performed. More precisely, multiple generalized linear regression (i.e., logistic with link logit) was carried out with covariates determined according to univariate results and to clinical relevance: ongoing cancer (vs. past cancer), male (vs. female), age, body mass index, education (high vs. low). Akaike Information Criterion (AIC) and Bayesian Information Criterion (BIC) were calculated and used as model diagnostics. Particular attention was paid to the study of multicollinearity and interactions between covariates. All two-by-two interactions between all covariates included in the final multivariate model were tested. Only the interaction between income and diploma was statistically significant. The relationships between continuous parameters were analyzed using Pearson and Spearman correlation coefficients. As the dimensions of the QLQ-C30 questionnaire were moderately to strongly correlated, each dimension (global health status, physical functioning, role functioning, emotional functioning, cognitive functioning, social functioning, fatigue, nausea and vomiting, pain, dyspnea, insomnia, appetite loss, constipation, diarrhea, and financial difficulties) was introduced separately in the multivariate model. The results were expressed as odds ratios and 95 Cis, and a forest plot was employed to present the results. As less than 5% of data were missing for the primary outcome, handling of missing data was not applied. However, sensitivity analyses were conducted to guarantee that these missing data did not influence the results, that is, representativeness was studied, especially for multivariable analyses, by comparing the sample with and without missing data for the main characteristics of the patients.

Statistical analysis was performed using Stata 15 (StataCorp, College Station, TX, USA). The tests were two-sided, with a type I error set at 5%. We chose to report all the individual *p*-values without applying any mathematical correction for the aforementioned tests comparing groups [21,22]. Specific attention was given to the magnitude of differences using Hedge’s effect sizes (ESs) and 95 CIs; they were interpreted according to the recommendations of Cohen, who defined the ES bounds as small (ES = 0.2), medium (ES = 0.5), and large (ES = 0.8) [23,24].

## 3. Results

### 3.1. Characteristics of Patients

**Figure 1:**
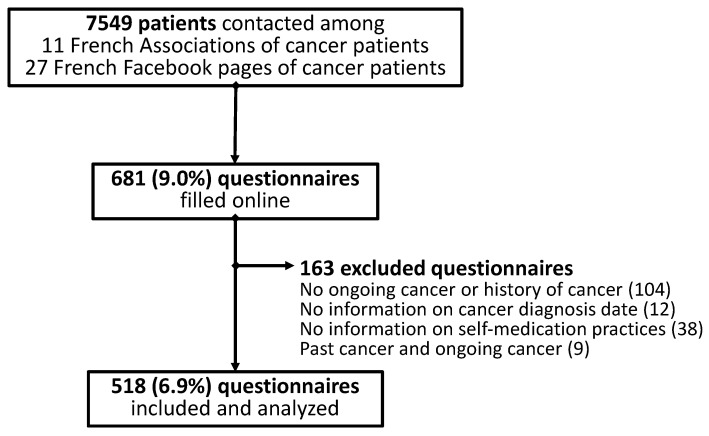
Flowchart of patients’ inclusion.

Six hundred and eighty-one patients answered the questionnaire, and five hundred and eighteen patients were included and analyzed in the study (Figure 1).

Among the 518 patients analyzed, 323 (62.4%) declared having an ongoing cancer (cancer patients) and 195 (37.6%) declared having been treated for a cancer (cancer survivors) at the time of the answer. Based on the 11 French associations and the 27 French Facebook pages of cancer patients, it was estimated that 7549 individuals had been contacted to answer the online questionnaire. The participation rate was about 6.9% (Figure 1). These 518 patients came from 76 of the 96 metropolitan departments in France (79.2%). The details of the included patients are presented in Table 1. Briefly, among all the patients analyzed, 80.4% were female, aged 52.5 ± 13.0 years, 42.2% had an education higher than a bachelor’s degree, and a monthly income of EUR 2000 (1400; 2500). Breast cancer was the most prevalent cancer, with a rate of 40.2% (Table 1). Patients with ongoing cancer declared they were being treated by ongoing oral anticancer medications (17.3%), injectable chemotherapy (25.1%), and radiotherapy (8.4%) (Table 1).

### 3.2. Self-Medication during and after Cancer

Among all the patients analyzed and at the time of answering, 292 (56.4%) (95 CI: 52.0; 60.7) declared they practiced self-medication, of which 192 (59.4%) (95 CI: 53.9; 64.8) were cancer patients and 100 (51.3%) (95 CI: 44.0; 58.5) were cancer survivors. No difference in patients’ characteristics was identified between self-medication and non-self-medication, whatever the group of patients (all the patients, cancer survivors, and cancer patients) (Table 1).

The products for self-medication used by the patients are presented in Table 2. Dietary supplements and pain medications were the main medications represented and used by more than half the patients. Other products were herbal medicines and digestive tract medications for a third of the patients, and essential oils, homeopathy, and anxiety and sleep disorder medications by a quarter of them (Table 2). Forty-one (14.0%) of the patients practicing self-medication declared using 5 classes of products, fifty-one (17.5%) 4 classes, sixty-four (21.9%) 3 classes, fifty-five (18.8%) 2 classes, and forty-five (15.4%) 1 class.

**Table 1:** Characteristics of the patients analyzed (all, cancer survivors, cancer patients, no self-medication (no SM), and self-medication (SM)). Results are presented by mean ± standard deviation, median (interquartile range), and number (percentage). * Other cancers for all the patients analyzed were hematological malignancies (11 (2.1%)), pancreas cancers (10 (1.9%)), head and neck cancers (8 (1.5%)), stomach cancers (4 (0.8%)), and skin cancers (4 (0.8%)). nc: not concerned.

Items	All	All			Cancer Survivors			Cancer Patients		
		No SM	SM	*p*-Value	No SM	SM	*p*-Values	No SM	SM	*p*-Values
Male Female	87 (19.6) 356 (80.4)	41 (22.3) 143 (77.7)	46 (17.8) 213(82.2)	0.28	17 (22.4) 59 (77.6)	17 (19.8) 69 (80.2)	0.70	24 (22.2) 84 (77.8)	29 (16.8) 144 (83.2)	0.28
Age (years)	52.5 ± 13.0	53.6 ± 13.6	51.8 ± 12.6	0.15	52.1 ± 14.5	51.5 ± 13.6	0.79	54.7 ± 12.9	51.9 ± 12.1	0.07
BMI (kg/m²)	25.5 ± 5.5	24.9 ± 5.0	25.9 ± 5.7	0.06	24.3 ± 4.2	25.3 ± 4.8	0.20	25.3 ± 5.6	26.2 ± 6.1	0.20
Education				0.18			0.77			0.14
No diploma or middle school	26 (5.9)	10 (5.4)	16 (6.2)		4 (5.2)	6 (7.1)		6 (5.6)	10 (5.8)	
BTEC first	63 (14.2)	35 (18.9)	28 (10.9)		13 (16.9)	12 (14.1)		22 (20.4)	16 (9.3)	
High school diploma	83 (18.7)	34 (18.4)	49 (19.0)		15 (19.5)	14 (16.5)		19 (17.6)	35 (20.2)	
Bachelor’s degree	84 (19.0)	30 (16.2)	54 (20.9)		10 (13.0)	17 (20.0)		20 (18.5)	37 (21.48)	
Higher than bachelor’s degree	187 (42.2)	76 (41.1)	111 (43.0)		35 (45.5)	36 (42.4)		41 (38.0)	75 (43.4)	
Income (EUR/month)	2000 (1400; 2500)	2000 (1400; 3000)	2000 (1400; 2500)	0.41	1900 (1400; 2500)	1900 (1390; 2400)	0.58	2000 (1400; 3000)	2.0 (1.4; 2.5)	0.51
Cancer diagnosis (years)	3.0 (1.0; 5.0)	3.0 (1.0; 5.0)	2.5 (1.0; 5.0)	0.75	3.0 (2.0; 5.0)	3.5 (2.0; 7.5)	0.45	2.0 (1.0; 5.0)	2.0 (1.0; 4.0)	0.85
Type of cancer *				0.21			0.76			0.07
Breast	208 (40.2)	82 (36.3)	126 (43.2)		32 (33.7)	39 (39.0)		50 (38.2)	87 (45.3)	
Kidney	52 (10.0)	26 (11.5)	26 (8.9)		4 (4.2)	7 (7.0)		22 (16.8)	19 (9.9)	
Prostate	39 (7.5)	16 (7.1)	23 (7.9)		11 (11.6)	9 (9.0)		5 (3.8)	14 (7.3)	
Ovary	35 (6.8)	10 (4.4)	25 (8.6)		4 (4.2)	9 (9.0)		6 (4.6)	16 (8.3)	
Lung	33 (6.4)	15 (6.6)	18 (6.2)		2 (2.1)	2 (2.0)		13 (9.9)	16 (8.3)	
Thyroid	32 (6.2)	15 (6.6)	17 (5.8)		7 (7.4)	5 (5.0)		8 (6.1)	12 (6.3)	
Cervix	24 (4.6)	13 (5.8)	11 (3.8)		11 (11.6)	9 (9.0)		2 (1.5)	2 (1.0)	
Bladder	24 (4.6)	15 (6.6)	9 (3.1)		5 (5.3)	3 (3.0)		10 (7.6)	6 (3.1)	
Colorectal	22 (4.3)	11 (4.9)	11 (3.8)		5 (5.3)	9 (9.0)		6 (4.6)	2 (1.0)	
Anticancer therapies (ongoing)					nc	nc	nc			
No therapy	46 (8.9)	24 (10.6)	22 (7.5)	0.28				24 (18.3)	22 (11.5)	0.10
Surgery	14 (2.7)	6 (2.7)	8 (2.7)	1.00				6 (4.8)	8 (4.2)	1.00
Oral anticancer medications	56 (10.8)	23 (10.2)	33 (11.3)	0.78				23 (17.6)	33 (17.2)	1.00
Injectable chemotherapy	81 (15.6)	29 (12.8)	52 (17.8)	0.14				29 (22.1)	52 (27.1)	0.36
Radiotherapy	27 (5.2)	12 (5.3)	15 (5.1)	1.00				12 (9.2)	15 (7.8)	0.69
Anticancer therapies (past)										
No therapy	24 (4.6)	7 (3.1)	17 (5.8)	0.21	3 (1.5)	1 (1.01)	1.00	6 (4.6)	15 (7.8)	0.36
Surgery	321 (62.0)	142 (62.8)	179 (61.3)	0.78	65 (68.4)	72 (72.0)	0.64	77 (58.8)	107 (55.7)	0.65
Oral anticancer medications	74 (14.3)	29 (12.8)	45 (15.4)	0.45	7 (7.4)	8 (8.0)	1.00	22 (16.8)	37 (19.3)	0.66
Injectable chemotherapy	252 (48.7)	110 (48.7)	142 (48.6)	1.00	51 (53.7)	49 (49.0)	0.57	59 (45.0)	93 (48.4)	0.57
Radiotherapy	253 (48.8)	103 (45.6)	150 (51.4)	0.22	57 (60.0)	65 (65.0)	0.55	46 (35.1)	85 (44.3)	0.11

The practice of self-medication to manage the adverse effects of anticancer therapies was declared by 183 (63.8%) of all the patients, 54 (55.7%) cancer survivors, and 129 (67.9%) cancer patients. The most frequent indications declared by the patients for the management of adverse effects of anticancer therapies were pain, fatigue, anxiety and/or stress, nausea and/or vomiting, and sleep disorders (Table 3). Among the patients with ongoing cancer, 84 (43.8%) declared using self-medication to improve the efficacy of anticancer therapies.

For all the patients, the places of purchase for the products were in community pharmacies (250 (85.6%)), the internet (69 (23.6%)), malls (19 (6.5%)), and other places (25 (8.6%)).

Patients declared that they received counsel for self-medication mainly from general practitioners (105 (36.0%)) and from community pharmacists (94 (32.2%)). Other sources of council were oncologists (77 (26.4%)), the internet (63 (21.6%)), friends (46 (15.8%)), and family (31 (10.6%)).

Among all the patients, 63 (21.6%) patients declared that they did not disclose any self-medication practice to health professionals involved in their management, likewise for 25 (25.0%) cancer survivors and 38 (19.8%) cancer patients. For the latter, the health professionals informed were oncologists (114 (59.4%)), general practitioners (88 (45.8%)), and pharmacists (35 (18.2%)).

Among patients with ongoing cancer, most of the patients believed that self-medication could not lead to drug interactions with anticancer therapies (163 (84.9%) answered no). Likewise, most of the patients thought that the practice of self-medication could not be related to adverse effects (all the patients: 247 (84.6%) answered no); this was also the case for 85 cancer survivors (85.0%) and 162 (84.4%) cancer patients.

### 3.3. Self-Medication, Quality of Life and Symptoms

Regarding HRQoL and symptoms, patients practicing self-medication had lower HRQoL scores for the social functioning dimension, and higher scores for pain and insomnia, in comparison to patients not practicing self-medication (Table 4). Patients practicing self-medication also reported higher scores for financial difficulties in comparison to those not practicing self-medication (Table 4).

When looking at the products of self-medication, all the dimensions of HRQoL (global health status, physical, role, emotional, cognitive, and social functioning) were decreased in patients taking pain medications in comparison to patients not using this self-medication class. Self-pain medications were also associated with high scores for fatigue, pain, and insomnia (Table 5). Digestive tract medications were associated with a decrease in the following HRQoL dimensions: role, emotional, cognitive, and social functioning, and with an increase in the following symptom dimensions: fatigue, nausea and vomiting, dyspnea, appetite loss, and diarrhea. Digestive tract medications were also associated with financial difficulties (Table 6). Anxiety and sleep disorder medications were associated with a decrease in the emotional and cognitive functioning dimensions. This class of self-medication was also associated with an increase in fatigue, pain, insomnia and constipation dimensions (Table 7). Mouth and throat medications were associated with a decrease in cognitive and social functioning dimensions, and an increase in financial difficulties (Table 7). Venous disorder medications were associated with an increase in dyspnea, insomnia, and constipation symptoms (Table 7). Patients taking dietary supplements, herbal therapies or essential oils had few modifications of QLQ-C30 dimensions (Table 5 and Table 6). Finally, patients taking homeopathy had no difference of HRQoL and symptom scores in comparison to patients not taking homeopathy (Table 6).

**Figure 2:**
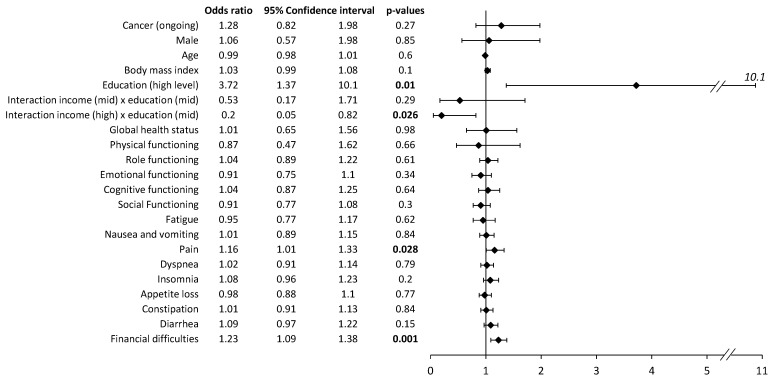
Multivariate analysis of self-medication (vs. no self-medication) at the time of the survey. The multivariate analysis included all the variables presented. The multivariate analysis was repeated independently for each item of the QLQ-C30 questionnaire. The results present odds ratios, 95% confidence intervals, and *p*-values. Bold *p*-values are significant (*p* < 0.05).

The multivariate analysis of self-medication revealed no impact of patients’ characteristics except for patients with higher education levels, who performed more self-medication than patients with lower education levels (Figure 2). However, there were interactions between education and income. Patients with high incomes and mid-level educations practiced less medication than others. Regarding HRQoL and symptoms, self-medication practices were associated with pain symptoms and financial difficulties (Figure 2). Multivariate analysis was conducted on 371 complete case patients. The representativeness of this sample was analyzed for self-medication, cancer (ongoing yes/no), gender, age, BMI, education, and income. No significant difference was highlighted.

## 4. Discussion

In this nationwide cross-sectional study, half of the patients practiced self-medication (59.4% with an ongoing cancer and 51.3% with a past cancer). None of the patients’ characteristics (oncology and socio-demographics) were associated with the self-medication practice, except for patients with higher educations (multivariate analysis). The most frequent products used for self-medication, regardless of the cancer status (ongoing or past cancer), were dietary supplements, pains medications, herbal medicines, and digestive tract medications. Two-thirds of patients declared they practiced self-medication to manage adverse effects of anticancer therapies, and the leading indications reported were pain, fatigue, anxiety and/or stress, nausea and/or vomiting, and sleep disorders. Forty-four percent of patients with an ongoing cancer declared they practiced self-medication to improve the efficacy of anticancer therapies. Community pharmacies were the main place where patients obtained their products for self-medication. The main sources of counsel were general practitioners and community pharmacists. A fifth of patients practicing self-medication did not disclose their practice to their health professionals. Most patients (85% answered no) were quite confident that self-medication cannot lead to drug interactions or adverse effects. Patients using self-medication had little or no alteration of their HRQoL, except a decrease in their social functioning. However, these patients had more pain and sleep disorders than patients who were not practicing self-medication. Finally, these patients also reported more financial issues related to the disease than patients who were not practicing self-medication. In multivariate analysis, pain and financial issues were the two leading factors associated with self-medication.

Self-medication in the older population (70–85 year) in France is about 48.7% [25]. In this cohort, self-medication was associated with sex (female) and higher education, but was not associated with lower HRQoL [25]. In comparison to a European study on the older population (>65 year), the prevalence of self-medication was about 23.6% [26]. This last study reported the same results regarding sex (female) and education [26]. However, comparisons of self-medication practices with other studies and countries should be interpreted with caution, because of the differences of local healthcare policies and advertising regulations [25]. In France, no drug (e.g., ibuprofen, paracetamol) can be sold outside community pharmacies [27].

Pain was strongly associated with self-medication, since patients who practiced self-medication with pain medications had a lower HRQoL (all dimensions), and worse symptoms of fatigue, pain, and insomnia. Besides pain, digestive tract disorders were also leading factors associated with self-medication, since these products for self-medication were also associated with a decrease in HRQoL (role, emotional, cognitive, and social functioning), and an increase in symptoms of fatigue, nausea/vomiting, dyspnea, appetite loss, and diarrhea. Likewise, medications for anxiety and sleep disorders were associated with a decrease in HRQoL (emotional and cognitive functioning), and an increase in fatigue, pain, insomnia, and constipation symptoms. These results underlined the most prevalent symptoms in cancer patients, as reported in advanced cancer patients, such as fatigue (18–76.3%), pain (18–75%), sleep problems (21.1–37.1%), dyspnea (19–67.3%), lack of appetite (13–80%), and gastrointestinal symptoms (12–45.1%) [28]. The study highlighted that pain is one of the main symptoms associated with self-medication during and after cancer. In a meta-analysis, pain was reported in 59% of patients undergoing cancer treatment (64% of patients with advanced disease, and 33% after curative treatment) [29]. It has been reported that in medical oncology outpatients, pain intensity was related to interference in daily activities, and that these patients subject to pain were insufficiently managed [30].

However, the relations between HRQoL/symptoms and self-medication practices are difficult to interpret. Two situations can be highlighted: on the one hand, patients with unmet medical needs inducing a self-medication practice for relief (e.g., high pain symptoms and use of pain medications), on the other hand, patients with relief induced by a self-medication practice (e.g., no modification of HRQoL or symptoms in patients using homeopathy). For homeopathy, the evidence of efficacy remains poor and biased [31], likewise for essential oils [32]. Considering the bibliographical data, it could be hypothesized that the decrease in HRQoL and/or the increase in symptoms leads patients to practice self-medication, and not the reverse.

As stated in the introduction, the use of CAM in adults with cancer in USA is highly prevalent, and has been reported to reach 87% [11]. In this latter study, the most frequently reported CAMs used were active product-based approaches (83.7%, including vitamins, minerals, non-mineral non-vitamin natural products, diet-based therapies, chelation therapy, diets, herbs, and tea), mind–body interventions (26.5%), manipulative and body-based therapies (22.6%), alternative medical systems (2.7%, including homeopathy, naturopathy, folk medicine, Ayurveda), and energy therapies (1.7%) [11]. CAM use was more prevalent among women, patients from 60 to 69 years of age, and those who had a higher level of education and were employed [11].

As reported in the present study, most of the patients were unaware of the potential risks (adverse effects or drug–drug interactions) associated with self-medication practices, such as OTC drugs, highlighting the need to inform patients about these risks [33,34]. Self-medication practices raises several issues of adverse effects and drug interactions that have been highlighted during the COVID-19 pandemic [35]. For example, in the specific context of cancer, several interactions between anticancer drugs and herbal medicines are well documented, such as enzymatic induction related to Saint John’s Wort or enzymatic inhibition related to grapefruit juice [36]. Herbal medicines are frequently used by cancer patients, ranging in prevalence from 19.7% to 69% [37,38,39,40], and patients taking herbal medicines have lower overall survival at 3 and 5 years than patients not taking herbal medicines [37], which underlines the unknown safety of these behaviors and self-medication practices. Moreover, self-medication practices can delay cancer diagnosis (e.g., gastrointestinal cancer [41,42], hematological malignancies [43]).

In the present study, more than half of the patients with an ongoing cancer disclosed their self-medication practice to their oncologist, which is somewhat lower than the results available in the literature. A systematic review reported that 77% of CAM users did not disclose their practice to medical practitioners [44]. The main concerns for the patients were about a negative response from the practitioners, the belief that the practitioner did not need to know about their CAM use, and the fact that the practitioner did not ask [44].

The definition of self-medication as such is also open to discussion, and the definition is clearly broader than simply taking medication on one’s own initiative. As presented in a scoping review on the definition of self-medication, the main common aspect of self-medication is individual action/behavior, which can be directed to oneself but also to another person (e.g., child) [6]. The authors proposed a broader scope including various concepts regarding the type of medications being used, the purpose of self-medication, the source of the medication, the severity of the condition, the type of individual, and the professional prescribing or recommending the medication [6]. A qualitative meta-synthesis described the perceptions and experiences of self-medication around the world, and identified five main themes, including cost-effectiveness, affectivity, the inefficiency of the healthcare system, previous experiences, and oversimplification, which could be classified into personal, social, organizational, and cultural categories [45].

In addition to the self-medication practices of cancer patients and survivors, it is also important to underline the role of health professionals in supporting these patients. Recently, based on a systematic review, a Canadian group proposed seven practice recommendations for health-care providers addressing CAM for cancer patients [46]. These practice recommendations included communicating, assessing, educating, decision coaching, documenting, active monitoring, and adverse event reporting [46]. The authors underlined the necessity for health-care providers to address CAM use as part of standard practice and to support evidence-informed decision making relating to CAM among individuals with cancer [46].

Finally, the present study underlined the patient’s economic burden of the disease, even if in France the cost of cancer care is fully paid by the national health insurance system. In 2019 in the USA, the national economic burden associated with cancer care for patients was estimated at $21.09 billion, made up of patient out-of-pocket costs of $16.22 billion and patient time costs (value of time spent traveling to and from health care, waiting for care, and receiving care) of $4.87 billion [47]. Self-care (including self-medication) has been demonstrated to substantially reduce healthcare costs (e.g., including allergic rhinitis/chronic urticaria, cold, migraine, cardiovascular disease, heartburn, vaginitis, and pain), by notably reducing physician visits [48]. However, in the specific context of cancer, this reduction in healthcare costs made possible by self-care remains to be demonstrated.

### Limitations of the Study

This type of cross-sectional study has a typical selection bias. The sample of patients was not representative of French cancer patients, with an over-representation of younger patients, females, and some type of cancers such as kidney, ovary, thyroid, cervix, and bladder cancers. In France, cancer incidence data shows a higher proportion of males over females (54% vs. 46%), with a median age of 68 years for males and 67 years for females. The most frequent cancers are breast (58,500 new cases) prostate (50,400 new cases), lung (46,363 new cases), and colorectal (43,336 new cases) [2]. Moreover, in this study, it was not possible to verify if the declared recourse to self-medication came from a medical prescription but was declared by the patient as self-medication (e.g., a medical prescription of paracetamol taken on demand). This study is one of the first to assess self-medication in cancer patients and survivors; however, precautions must be taken in the interpretation and generalization of the results, particularly for drivers of self-medication, for which further studies should be carried out.

## 5. Conclusions

In our sample of individuals, the practice of self-medication during and after cancer treatments is quite highly prevalent but does not seem to be higher than that of the overall French population. In comparison to the international scientific data, French cancer patients appear to be more open about disclosing their self-medication practices to health professionals. However, these cancer patients are quite confident about the safety of self-medication, thus emphasizing the need for health professionals to accompany and inform patients about their self-medication practices. For cancer patients, self-medication could be a strong marker of unmet medical needs, mainly relating to chronic pain, and associated disorders such as HRQoL impairment and psychological distress [49]. Finally, French health authorities should pay attention to the economic burden of self-medication practices for patients.

## Figures and Tables

**Table 2:** Products for self-medication used by all the patients, the cancer survivors and the cancer patients.

Products for Self-Medication	All N (%)	Cancer Survivors N (%)	Cancer Patients N (%)
Dietary supplements	175 (59.9)	66 (66.0)	109 (56.8)
Pain medications	171 (58.6)	64 (64.0)	107 (55.7)
Herbal medicines	103 (35.3)	40 (40.0)	63 (32.8)
Digestive tract medications	98 (33.6)	38 (38.0)	60 (31.3)
Essential oils	86 (29.5)	29 (29.0)	57 (29.7)
Homeopathy	80 (27.4)	28 (28.0)	52 (27.1)
Anxiety and sleep disorder medications	69 (23.6)	28 (28.0)	41 (21.4)
Mouth and throat medications	45 (15.4)	19 (19.0)	26 (13.5)
Venous disorder medications	26 (8.9)	7 (7.0)	19 (9.9)

**Table 3:** Indications for self-medication to manage the adverse effect of anticancer therapies, for all the patients, among patients with a past cancer, and among patients with an ongoing cancer.

Adverse Effects	All N (%)	Cancer Survivors N (%)	Cancer Patients N (%)
Pain	114 (39.0)	36 (36.0)	78 (40.6)
Fatigue	74 (25.3)	26 (26.0)	48 (25.0)
Anxiety and/or stress	63 (21.6)	23 (23.0)	40 (20.8)
Nausea and/or vomiting	61 (20.9)	18 (18.0)	43 (22.4)
Sleep disorders	52 (17.8)	20 (20.0)	32 (16.7)
Stomach aches	50 (17.1)	15 (15.0)	35 (18.2)
Hot flushes	48 (16.4)	21 (21.0)	27 (14.1)
Constipation	46 (15.8)	16 (16.0)	30 (15.6)
Diarrhea	42 (14.4)	9 (9.0)	33 (17.2)
Oral lesions	36 (12.3)	11 (11.0)	25 (13.0)
Nail and/or hair disorders	32 (11.0)	12 (12.0)	20 (10.4)
Skin disorders	32 (11.0)	11 (11.0)	21 (10.9)
Vaginal disorders	27 (9.3)	12 (12.0)	15 (7.8)
Depression	24 (8.2)	11 (11.0)	13 (6.8)
Sexual disorders	10 (3.4)	4 (4.0)	6 (3.1)
Respiratory disorders	8 (2.7)	1 (1.0)	7 (3.7)

**Table 4:** HRQoL and symptoms for all the patients according to self-medication (no self-medication (no SM), and self-medication (SM)). Bold effect sizes and *p*-Values are significant.

QLQ-C30	All	No SM	SM	Effect Size	*p*-Values
Global health status	60.6 ± 19.5	62.3 ± 20.8	59.2 ± 18.4	−0.06(−0.24; 0.13)	0.07
Physical functioning	77.3 ± 21.1	78.6 ± 21.4	76.3 ± 20.9	−0.04 (−0.22; 0.14)	0.12
Role functioning	59.0 ± 32.3	59.6 ± 33.9	58.5 ± 31.1	0.06 (−0.11; 0.24)	0.60
Emotional functioning	57.6 ± 28.8	60.2 ± 29.6	55.7 ± 28.1	−0.07 (−0.25; 0.12)	0.07
Cognitive functioning	64.3 ± 30.2	65.6 ± 31.3	63.3 ± 29.4	0.03 (−0.15; 0.21)	0.26
Social functioning	58.2 ± 32.1	63.0 ± 32.0	54.6 ± 31.8	**−0.14 (−0.32; 0.05)**	**0.003**
Fatigue	54.5 ± 29.3	53.1 ± 29.7	55.6 ± 29.0	0.08 (−0.10; 0.26)	0.33
Nausea and vomiting	16.7 ± 26.2	19.0 ± 29.8	14.9 ± 23.0	−0.02 (−0.20; 0.16)	0.74
Pain	42.0 ± 32.2	36.9 ± 31.2	46.0 ± 32.4	**0.22 (−0.04; 0.40)**	**0.001**
Dyspnea	30.7 ± 31.6	29.9 ± 32.2	31.2 ± 31.2	0.09 (−0.10; 0.27)	0.51
Insomnia	49.5 ± 36.8	43.4 ± 35.1	54.2 ± 37.5	**0.20 (−0.02; 0.38)**	**0.002**
Appetite loss	24.6 ± 33.6	25.2 ± 34.2	24.1 ± 33.2	−0.02 (−0.20; 0.16)	0.78
Constipation	29.1 ± 35.1	27.8 ± 34.7	30.1 ± 35.4	0.06 (−0.12; 0.24)	0.47
Diarrhea	20.2 ± 29.6	19.5 ± 30.8	20.8 ± 28.8	0.12 (−0.06; 0.31)	0.26
Financial difficulties	25.0 ± 34.2	19.2 ± 31.5	29.4 ± 35.5	**0.33 (0.15; 0.52)**	**<0.001**

**Table 5:** HRQoL and symptoms for all the patients according to self-medication with dietary supplements, pain medications, and herbal medicines. The results present the comparison between patients taking specific products for self-medication (no vs. yes and effect size (95 CI)). * *p* < 0.05, ** *p* < 0.01, *** *p* < 0.001. Bold effect sizes are significant.

QLQ-C30	Dietary Supplements	Pain Medications	Herbal Medicines
	Scores (No vs. Yes) Effect Size (95 CI)	Scores (No vs. Yes) Effect Size (95 CI)	Scores (No vs. Yes) Effect Size (95 CI)
Global health status	57.7 ± 18.8 vs. 60.1 ± 18.1 0.13 (−0,12; 0.38)	63.8 ± 18.3 vs. 56.0 ± 17.8 *** **−0.43 (−0.68; −0.18)**	58.5 ± 19.0 vs. 60.5 ± 17.2 0.11 (−0.14; 0.35)
Physical functioning	74.7 ± 20.8 vs. 77.4 ± 20.9 0.13 (−0.11; 0.37)	81.5 ± 18.0 vs. 72.7 ± 22.1 *** **−0.42 (−0.66; −0.18)**	75.3 ± 21.4 vs. 78.2 ± 20.0 0.14 (−0.11; 0.38)
Role functioning	54.4 ± 29.5 vs. 60.9 ± 31.8 0.21 (−0.03; 0.45)	67.1 ± 29.2 vs. 52.5 ± 31.1 *** **−0.48 (−0.72; −0.24)**	56.8 ± 31.8 vs. 61.4 ± 29.8 0.15 (−0.10; 0.39)
Emotional functioning	56.3 ± 28.0 vs. 55.3 ± 28.3 −0.04 (−0.29; 0.21)	65.2 ± 25.6 vs. 49.0 ± 28.0 *** **−0.60 (−0.85; −0.35)**	55.1 ± 29.2 vs. 56.6 ± 26.3 0.05 (−0.19; 0.30)
Cognitive functioning	62.1 ± 27.1 vs. 64.0 ± 30.8 0.06 (−0.18; 0.31)	68.5 ± 29.6 vs. 59.7 ± 28.9 ** **−0.30 (−0.54; −0.06)**	63.0 ± 30.6 vs. 63.8 ± 27.5 0.03 (−0.22; 0.27)
Social functioning	52.9 ± 31.6 vs. 55.6 ± 32.0 0.08 (−0.16; 0.33)	60.6 ± 32.0 vs. 50.3 ± 31.1 * **−0.33 (−0.57; −0.08)**	54.6 ± 32.9 vs. 54.4 ± 29.9 −0.01 (−0.25; 0.24)
Fatigue	57.1 ± 27.7 vs. 54.7 ± 29.9 −0.08 (−0.32; 0.16)	45.9 ± 28.3 vs. 62.4 ± 27.7 *** **0.59 (0.34; 0.83)**	56.8 ± 30.8 vs. 53.6 ± 25.8 −0.11 (−0.35; 0.14)
Nausea and vomiting	14.6 ± 22.0 vs. 15.1 ± 23.7 0.02 (−0.22; 0.26)	11.4 ± 19.2 vs. 17.4 ± 25.1 0.26 (0.02; 0.50)	16.1 ± 24.8 vs. 12.9 ± 19.7 −0.14 (−0.38; 0.11)
Pain	49.2 ± 29.8 vs. 44.0 ± 33.9 −0.16 (−0.40; 0.08)	34.2 ± 30.0 vs. 54.1 ± 31.6 *** **0.64 (0.39; 0.88)**	45.8 ± 33.6 vs. 46.2 ± 30.4 0.01 (−0.23; 0.26)
Dyspnea	32.4 ± 31.5 vs. 30.6 ± 31.0 −0.06 (−0.30; 0.19)	28.9 ± 32.1 vs. 32.9 ± 30.5 0.13 (−0.11; 0.37)	32.4 ± 31.5 vs. 29.3 ± 30.6 −0.10 (−0.34; 0.15)
Insomnia	54.0 ± 37.4 vs. 54.3 ± 37.7 0.01 (−0.24; 0.25)	43.2 ± 37.1 vs. 61.9 ± 35.9 *** **0.51 (0.27; 0.76)**	52.4 ± 38.7 vs. 57.3 ± 35.2 0.13 (−0.11; 0.38)
Appetite loss	29.8 ± 35.8 vs. 20.5 ± 31.1 * **−0.28 (−0.53; −0.04)**	19.3 ± 29.9 vs. 27.4 ± 35.0 0.24 (0.00; 0.48)	26.2 ± 35.1 vs. 20.5 ± 29.4 −0.17 (−0.42; 0.07)
Constipation	26.6 ± 33.9 vs. 32.2 ± 36.1 0.16 (−0.09; 0.40)	26.8 ± 33.9 vs. 32.3 ± 36.3 0.15 (−0.09; 0.39)	30.8 ± 35.5 vs. 28.7 ± 35.3 −0.06 (−0.30; 0.18)
Diarrhea	26.6 ± 32.3 vs. 17.4 ± 25.9 −0.32 (−0.57; −0.07)	17.9 ± 28.2 vs. 22.9 ± 29.0 0.17 (−0.07; 0.42)	24.2 ± 30.5 vs. 15.1 ± 24.5 * **−0.32 (−0.57; −0.06)**
Financial difficulties	31.3 ± 34.8 vs. 28.3 ± 35.9 −0.08 (−0.33; 0.17)	25.1 ± 34.2 vs. 32.3 ± 36.1 0.20 (−0.04; 0.45)	29.1 ± 33.4 vs. 29.9 ± 38.9 0.02 (−0.23; 0.27)

**Table 6:** HRQoL and symptoms for all the patients according to self-medication with digestive tract medications, essential oils, and homeopathy. The results present the comparison between patients taking specific products for self-medication (no vs. yes and effect size (95 CI)). * *p* < 0.05, ** *p* < 0.01, *** *p* < 0.001. Bold effect sizes are significant.

QLQ-C30	Digestive Tract Medications	Essential Oils	Homeopathy
	Scores (No vs. Yes) Effect Size (95 CI))	Scores (No vs. Yes) Effect Size (95 CI)	Scores (No vs. Yes) Effect Size (95 CI)
Global health status	61.2 ± 17.8 vs. 55.4 ± 19.3 0.31 (−0.57; −0.06)	59.0 ± 18.7 vs. 59.8 ± 17.7 −0.04 (−0.22; 0.30)	58.6 ± 18.9 vs. 60.7 ± 17.1 −0.11 (−0.15; 0.38)
Physical functioning	77.8 ± 20.3 vs. 73.5 ± 21.8 0.21 (−0.45; 0.04)	76.4 ± 21.2 vs. 76.2 ± 20.2 0.01 (−0.26; 0.25)	75.6 ± 21.3 vs. 78.2 ± 19.7 −0.12 (−0.14; 0.38)
Role functioning	61.8 ± 30.1 vs. 51.6 ± 32.2 * **0.33 (−0.58; −0.08)**	59.5 ± 31.2 vs. 56.0 ± 30.9 0.12 (−0.37; 0.14)	57.9 ± 31.0 vs. 59.8 ± 31.6 −0.06 (−0.20; 0.32)
Emotional functioning	59.0 ± 27.6 vs. 49.0 ± 28.2 ** **0.36 (−0.61; −0.10)**	56.8 ± 27.9 vs. 53.1 ± 28.8 0.13 (−0.39; 0.13)	54.2 ± 29.6 vs. 59.2 ± 24.0 −0.18 (−0.09; 0.44)
Cognitive functioning	66.2 ± 28.2 vs. 57.6 ± 31.1 * **0.29 (−0.55; −0.04)**	66.0 ± 28.0 vs. 57.2 ± 31.9 * **0.30 (−0.56; −0.04)**	62.3 ± 30.0 vs. 65.8 ± 28.0 −0.12 (−0.15; 0.38)
Social functioning	57.3 ± 32.0 vs. 49.3 ± 30.9 * **0.25 (−0.51; 0.00)**	57.2 ± 32.3 vs. 48.3 ± 29.8 * **0.28 (−0.54; −0.02)**	53.8 ± 32.1 vs. 56.4 ± 31.1 −0.08 (−0.19; 0.34)
Fatigue	52.6 ± 29.3 vs. 61.8 ± 27.6 * **−0.32 (0.07; 0.57)**	54.1 ± 29.7 vs. 59.1 ± 27.2 −0.17 (−0.08; 0.43)	55.6 ± 29.9 vs. 55.7 ± 26.8 −0.00 (−0.26; 0.26)
Nausea and vomiting	11.1 ± 19.4 vs. 22.6 ± 27.6 *** **−0.52 (0.26; 0.77)**	14.1 ± 22.8 vs. 16.7 ± 23.6 −0.11 (−0.15; 0.36)	15.0 ± 23.7 vs. 14.7 ± 21.5 0.01 (−0.27; 0.25)
Pain	43.3 ± 31.5 vs. 51.3 ± 33.7 −0.25 (−0.00; 0.50)	43.9 ± 32.3 vs. 50.6 ± 32.4 −0.21 (−0.05; 0.46)	44.9 ± 32.1 vs. 48.7 ± 33.2 −0.12 (−0.14; 0.38)
Dyspnea	28.5 ± 30.5 vs. 36.7 ± 32.0 * −0.26 (0.01; 0.51)	29.6 ± 30.7 vs. 35.0 ± 32.0) −0.17 (−0.09; 0.43)	31.6 ± 31.6 vs. 30.3 ± 30.2 0.04 (−0.31; 0.22)
Insomnia	51.0 ± 38.6 vs. 60.5 ± 34.6 −0.25 (0.00; 0.50)	52.4 ± 37.4 vs. 58.3 ± 37.6 −0.16 (−0.10; 0.42)	54.7 ± 38.4 vs. 53.0 ± 35.4 0.05 (−0.31; 0.22)
Appetite loss	20.7 ± 31.6 vs. 30.8 ± 35.4 * **−0.31 (0.05; 0.56)**	24.4 ± 33.0 vs. 23.4 ± 33.8 0.03 (−0.29; 0.23)	22.2 ± 32.6 vs. 29.0 ± 34.3 −0.21 (−0.06; 0.47)
Constipation	28.4 ± 35.4 vs. 33.3 ± 35.3 −0.14 (−0.11; 0.39)	29.0 ± 35.2 vs. 32.5 ± 35.8 −0.10 (−0.16; 0.36)	31.3 ± 36.7 vs. 26.9 ± 31.8 0.12 (−0.39; 0.14)
Diarrhea	16.2 ± 27.4 vs. 30.0 ± 29.3 *** **−0.49 (0.23; 0.75)**	22.7 ± 30.5 vs. 16.5 ± 23.8 0.22 (−0.48; 0.05)	21.0 ± 29.3 vs. 20.3 ± 27.7 0.02 (−0.29; 0.24)
Financial difficulties	24.5 ± 32.7 vs. 39.0 ± 38.7 ** **−0.41 (0.16; 0.67)**	29.0 ± 35.7 vs. 30.3 ± 35.1 −0.04 (−0.23; 0.30)	27.7 ± 34.7 vs. 33.8 ± 37.2 −0.17 (−0.10; 0.44)

**Table 7:** HRQoL and symptoms for all the patients according to self-medication with anxiety and sleep disorders medications, mouth and throat medications, venous disorder medications. The results present the comparison between patients taking specific products for self-medication (no vs. yes and effect size (95 CI)). * *p* < 0.05, ** *p* < 0.01, *** *p* < 0.001. Bold effect sizes are significant.

QLQ-C30	Anxiety and Sleep Disorder Medications	Mouth and Throat Medications	Venous Disorder Medications
	Scores (No vs. Yes) Effect Size (95 CI)	Scores (No vs. Yes) Effect Size (95 CI)	Scores (No vs. Yes) Effect Size (95 CI)
Global health status	59.1 ± 18.8 vs. 59.8 ± 17.0 −0.04 (−0.24; 0.33)	59.4 ± 19.2 vs. 58.1 ± 13.2 0.07 (−0.41; 0.27)	59.3 ± 18.6 vs. 59.0 ± 17.0 0.01 (−0.43; 0.41)
Physical functioning	76.8 ± 20.2 vs. 74.9 ± 23.2 0.09 (−0.37; 0.18)	76.1 ± 21.5 vs. 77.6 ± 17.3 −0.07 (−0.26; 0.40)	76.4 ± 20.9 vs. 76.0 ± 21.3 0.02 (−0.43; 0.39)
Role functioning	60.0 ± 30.8 vs. 53.3 ± 31.8 0.22 (−0.49; 0.06)	58.6 ± 31.4 vs. 57.5 ± 29.5 0.03 (−0.36; 0.29)	58.3 ± 31.2 vs. 60.0 ± 30.8 −0.05 (−0.36; 0.46)
Emotional functioning	58.78 ± 26.6 vs. 45.3 ± 30.7 ** **0.49 (−0.77; −0.20)**	55.6 ± 28.6 vs. 56.2 ± 25.6 −0.02 (−0.31; 0.36)	56.0 ± 28.4 vs. 52.0 ± 25.5 0.14 (−0.56; 0.27)
Cognitive functioning	65.5 ± 28.8 vs. 56.2 ± 30.5 * **0.32 (−0.60; −0.03)**	64.6 ± 29.7 vs. 55.8 ± 26.8 * **0.30 (−0.63; 0.04)**	63.9 ± 29.4 vs. 56.9 ± 29.5 0.24 (−0.66; 0.18)
Social functioning	54.9 ± 31.9 vs. 53.3 ± 31.7 0.05 (−0.34; 0.23)	56.1 ± 32.2 vs. 45.8 ± 28.4 * **0.32 (−0.66; 0.01)**	54.4 ± 32.1 vs. 56.3 ± 29.4 −0.06 (−0.36; 0.48)
Fatigue	53.6 ± 29.4 vs. 62.1 ± 26.9 * **−0.29 (0.02; 0.57)**	55.3 ± 29.6 vs. 57.4 ± 25.6 −0.07 (−0.26; 0.40)	55.0 ± 29.3 vs. 62.0 ± 26.4 −0.24 (−0.17; 0.65)
Nausea and vomiting	14.8 ± 22.9 vs. 15.4 ± 23.8 −0.03 (−0.25; 0.30)	14.1 ± 22.2 vs. 19.4 ± 27.0 −0.23 (−0.10; 0.56)	14.9 ± 23.6 vs. 15.3 ± 17.3 −0.02 (−0.39; 0.43)
Pain	43.5 ± 32.0 vs. 53.8 ± 32.9 * **−0.32 (0.04; 0.60)**	45.3 ± 32.3 vs. 49.6 ± 33.2 −0.13 (−0.20; 0.46)	45.0 ± 32.7 vs. 55.3 ± 28.8 −0.32 (−0.09; 0.73)
Dyspnea	30.0 ± 30.7 vs. 35.5 ± 32.7 −0.18 (−0.11; 0.46)	30.3 ± 31.5 vs. 36.6 ± 28.7 −0.20 (−0.13; 0.53)	29.3 ± 29.5 vs. 51.4 ± 40.5 ** **−0.72 (0.30; 1.14)**
Insomnia	47.7 ± 37. vs. 75.5 ± 29.2 *** **−0.78 (0.49; 1.07)**	52.6 ± 37.6 vs. 63.4 ± 35.6 −0.29 (−0.04; 0.62)	52.6 ± 38.0 vs. 70.7 ± 27.8 * **−0.49 (0.07; 0.90)**
Appetite loss	23.8 ± 33. vs. 25.0 ± 33.1 −0.04 (−0.24; 0.31)	22.8 ± 32.8 vs. 31.0 ± 34.8 −0.24 (−0.08; 0.57)	23.3 ± 32.5 vs. 32.0 ± 39.1 −0.26 (−0.15; 0.67)
Constipation	27.9 ± 35. vs. 36.9 ± 35.4 * **−0.25 (−0.02; 0.53)**	28.8 ± 35.1 vs. 37.3 ± 36.2 −0.24 (−0.09; 0.57)	28.8 ± 35.1 vs. 42.7 ± 36.7* **−0.39 (−0.02; 0.80)**
Diarrhea	22.5 ± 30.3 vs. 15.3 ± 22.4 0.25 (−0.54; 0.04)	20.4 ± 29.0 vs. 23.3 ± 27.4 −0.10 (−0.23; 0.44)	21.0 ± 29.0 vs. 18.8 ± 26.3 0.08 (−0.50; 0.35)
Financial difficulties	28.0 ± 34.7 vs. 33.8 ± 37.8 −0.16 (−0.12; 0.45)	26.8 ± 34.5 vs. 44.4 ± 37.7 ** **−0.50 (0.16; 0.85)**	29.4 ± 35.9 vs. 29.2 ± 31.6 0.01 (−0.43; 0.41)

## Data Availability

The data will be made available upon request to the corresponding author.

## Supplementary Materials

The following supporting information can be downloaded at: https://www.mdpi.com/article/10.3390/cancers15123190/s1.
