# Supplementary material for: Self-Medication during and after Cancer: A French Nation-Wide Cross-Sectional Study
Source: Cancers (Basel). 2023 Jun 15;15(12):3190. doi: 10.3390/cancers15123190 (PMC10296279; doi:10.3390/cancers15123190)
Supplement: Supplementary file 1 [file cancers-15-03190-s001.zip › cancers-2406692-supplementary.pdf]

List of the 11 associations of cancer patients

Association réseaux papillon
Association nationale des malades du cancer de la prostate (ANAMACAP)
Europa donna
ARTUR Association pour la recherche sur les tumeurs du rein
Geneticancer
Vivre comme avant
Jeune et rose
Les zuros
De l'air
Patients en réseaux
Toujours femme

List of the 27 Facebook pages

Cancer du sein (https://www.facebook.com/groups/163977141139047)
Cancer l'annonce, les traitements, … (https://www.facebook.com/groups/1059704954172777)
Ensemble contre le cancer (https://www.facebook.com/groups/198824690930)
Cancer du sein (https://www.facebook.com/groups/1480053655572660)
Tous contre le cancer (https://www.facebook.com/groups/304700023052435)
Cancer de l'ovaire, parlons-en en toute liberté (https://www.facebook.com/groups/2315125465471549)
Campagne cancer du sein (https://www.facebook.com/groups/849967579116188)
Le cancer et moi (https://www.facebook.com/groups/877264495762167)
Prévention cancer du sein (https://www.facebook.com/groups/487349962627597)
Cancer alimentation et cie (https://www.facebook.com/groups/524914372195614)
Le cancer et les proches (https://www.facebook.com/groups/856275948066338)
La vie après le cancer (https://www.facebook.com/groups/1581511005422427)
Espoir (pour lutter contre le cancer du poumon) (https://www.facebook.com/groups/38037140484)
Le cancer de la thyroïde (https://www.facebook.com/groups/227789050615254)
Communauté de soutien aux victimes du cancer (https://www.facebook.com/groups/763337773726635)
La vie en rose soutien cancer du sein (https://www.facebook.com/groups/lavieenrosessoutiencancerdusein)
Cancer pancréas soutiens et encouragements (https://www.facebook.com/groups/283014431877492)
Groupe, chaine en soutien aux personnes victimes de cancer et des aidants (https://www.facebook.com/groups/170405290410707)
Les roses : soutiens, infos et humour autour du cancer du sein https://www.facebook.com/groups/441380866631113)
Cancer du pancréas et du colon (https://www.facebook.com/groups/1639452816336238)
Cancer du col de l'utérus bis (https://www.facebook.com/groups/321466854568734)
A toutes celles qui ont ou ont eu le cancer du col de l'utérus (https://www.facebook.com/groups/35906114247)
Mélanome France (https://www.facebook.com/groups/melanomamatesfr)
Cancer primitif du foie (https://www.facebook.com/groups/782693335151521)
Cancer du rectum (https://www.facebook.com/groups/196698777899332)
Cancer du foie et thrombose portale (https://www.facebook.com/groups/246729702712628)
Truc et conseils pour chimiothérapie (https://www.facebook.com/groups/566968916977248)
